# A Plea for Purity

**Published:** 1886-01

**Authors:** 


### Correspondence.

### A PLEA FOR PURITY.

*Editors Atlanta Medical and Surgical Journal:*

A case of self-mutilation, in my immediate neighborhood, during a fit of insanity brought on by the practice of masturbation, impels me to write a few lines, hoping through the columns of your excellent journal to reach the ears of a large number of medical men that they may be induced to use their influence (in a way which I fear too many now neglect or slight). I refer to the influence every practitioner has to warn in every available way against the too common practice of indulging in low and degrading (though not always obscene) talk. In olden time "a man was famous according as he had lifted up axes upon the thick trees." I see no reason why a man should be less famous in these modern days, who does fearlessly and unhesitatingly attack the too common sins of society. The whole country honors an earnest minister of the gospel who dares to denounce fashionable sins. Why should they regard with less respect the worthy M. D., who, with equal earnestness and fearlessness, denounces with withering scorn all those whose own souls are so sunken in the filth of polluting crime or imagination, that they find pleasure in scattering their obscene thoughts in the face of those whom they associate with, seemingly to bring others to the same low degree as themselves.

The minister is supposed to fight for all that is "good and true and beautiful," and against everything that is impure and unholy in the moral world, while the doctor is supposed to wage war against everything that mars the body in health or beauty and to labor to restore that health or beauty wherever it has been lost or impaired.

May we not hope that every physician to whom this plea shall come will begin from this time (if not before) to lift up his voice in no uncertain sound against the vulgar talk and obscene innuendoes that occupy too much time among the young men, and too often older ones, as they seem to forget that the thought is the father of the act, and the conversation is a step toward the commission of the crime. For does not the blessed Saviour say, "Out of the heart proceed evil thoughts, murders, adulteries, fornications, etc." And I do not think it will be out of place to warn the colored people against the same filthy sin. They do an immense deal of dirty talk when they are together, and their animal passions are aroused and stimulated until they find an opportunity (without fear of detection, as they think), and they commit the crime for which a justly indignant crowd takes summary vengeance on the trespasser. Now, we know not whose wife or daughter will be the next victim of this beastly passion (the inevitable result of so much filthy conversation), so that the safety of our homes demands that we should not only set our face as a flint against this nuisance, but take every suitable occasion to sound the warning by speaking against it, both to white and colored.

I see no reason why the physician may not (or rather should not) be a positive factor for good, moral as well as physical, to the community in which he moves, and he has so much the advantage of the clergy that he moves among the people with so much more familiarity in his intercourse, and hence has more opportunities to speak a word for *the pure*, and, besides, his word will have more weight if he takes the pains to show the intimate connection that exists between the purity of spirit and conversation, and the health and sanity of body, and that it is those whose minds allow their imaginations to harbor all sorts of forbidden thoughts are the very ones that fall into the temptation of committing the crime that brings on them the punishment of the law or the mob.

SENEX.

